# Bibliometric analysis of oncolytic virus research, 2000 to 2018

**DOI:** 10.1097/MD.0000000000016817

**Published:** 2019-08-30

**Authors:** Yidi Zou, Yong Luo, Jun Zhang, Ningshao Xia, Guowei Tan, Chenghao Huang

**Affiliations:** a State Key Laboratory of Molecular Vaccinology and Molecular Diagnostics, National Institute of Diagnostics and Vaccine Development in Infectious Diseases, School of Public Health, Xiamen University; b Department of Neurosurgery, First Affiliated Hospital of Xiamen University, Xiamen, China.

**Keywords:** bibliometric, citation, H-index, oncolytic virus

## Abstract

**Background:**

Accumulating evidence in the last decade has pointed to the effectiveness of oncolytic virus in the treatment of a variety of cancer types in preclinical or clinical studies, showing high potency and low toxicity compared to conventional treatments. To track this research trend and highlight future directions, we conducted a bibliometric analysis of oncolytic virus research to date.

**Methods:**

Relevant studies were obtained from the Web of Science Core Collection between January 2000 and December 2018. Data were collected in terms of the number of publications, country, journal of publication, journal scope, author, and keywords or topics. Analysis and visual representation of the data were performed with CiteSpace V.

**Results:**

The trend in publications related to oncolytic virus showed a dramatic increase, from 10 publications in 2000 to 199 publications in 2018. The United States clearly dominates this field (981 publications, 52.770%), followed by Canada (244, 13.125%) and China (205, 11.027%). The top 15 academic journals account for over one third of the total publications on oncolytic virus research (724, 38.95%). Most of the related papers were published in journals with a focus on biology, medicine, immunology, medicine, molecular biology, and clinical perspectives, as represented by the dual-map overlay. The most highly cited papers were published in journals in the fields of nursing, molecular biology, general biology, genetics, health, and medicine. Over 1300 institutions have focused their attention on oncolytic virus research to date, and cooperation among mainstream institutions is common.

**Conclusion:**

The global field of oncolytic virus research has expanded at a rapid pace from 2000 to 2018. There is no doubt that North America currently has the most powerful impact on the field with respect to both productivity and contribution. However, European and some East Asian institutions are also prominent in this field. Overall, this bibliometric study identifies the top 4 hotspots in oncolytic virus research: T-cells, vaccinia virus, dendritic cells, and apoptosis. Thus, further research focuses on these topics may be more helpful to promote the clinical translation of this treatment strategy to bring a benefit to cancer patients in the near future.

## Abstract 2

Supplemental Digital Content is available in the text

## 1 Introduction

Oncolytic viruses (OVs) are viruses that can be specifically edited or selected to target their replication in cancer cells for their selective destruction.^[1]^ OVs are also very effective at inducing immune responses to the infected tumor cells, thereby enhancing the ability of the body to fight the cancer.^[2]^ Although the cancer cell-killing ability of viruses has been recognized for over a century, the effectiveness of OVs in the treatment of cancer patients has only recently been clinically documented.^[3–5]^ In 2015, Imlygic, a herpes virus-based therapeutic, broke through the “glass ceiling” of this strategy and was the first OV to be approved by the FDA for cancer treatment.^[6]^ Since then, a large number of OV-based drugs have entered clinical trials. Two OVs are currently in phase III trials, at least 49 are in phase I or II trials, and these numbers are expected to increase dramatically in the near future along with research progress in this field.^[7]^

Thus, OVs are now a potent weapon in the armory of the oncologist, and numerous academic journals have started to pay attention to this emerging research field. Indeed, the number of papers related to OVs has been on the rise in the last decade in particular. Thus, this is an important period of time to evaluate the quantitative and qualitative value of this body of literature from a scientific perspective. Bibliometrics is a novel method for assessing recent research activities. In particular, bibliometrics can characterize the trends of research on a given subject, which can help to inform clinical guidelines and govern policy making.^[8]^

Therefore, in this study, we adopted a bibliometrics approach to analyze the trends in OV publications, which were obtained from the Web of Science (Thomson Reuters Company) database from 2000 to 2018. Detailed analysis of these trends can provide a better overview of the changes of this field and offer perspective on the most important hotspots worthy of further exploration.

## 2 Methods

### 2.1 Data sources and strategy of data retrieval

All articles were retrieved from the Web of Science Core Collection (WoSCC). The WoSCC is known to harbor relatively reliable data and can provide abundant information for analysis.^[9]^ Although the WoSCC offers a variety of publication types, this study has only selected peer-reviewed research articles or review articles, considering the reliability of the articles. The publications from the WoSCC has been open to the researchers since 1975, but only 17 articles published from 1975 to 2000 met the criteria. Thus, the articles published from 2000 to 2018 was retrieved for further study.

**Figure 1:**
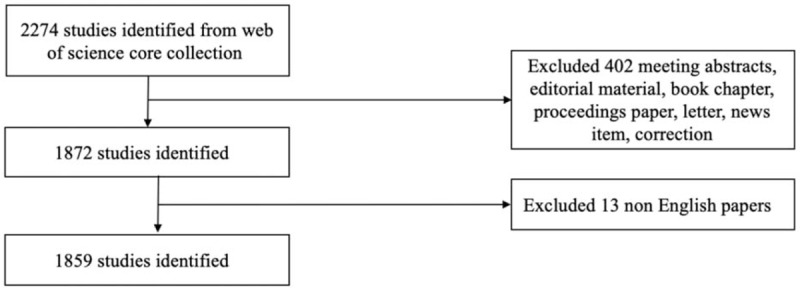
The inclusion criteria for paper selection.

The detailed data retrieval strategy and inclusion procedure of this study is summarized in Figure 1. In brief, we specified the search terms as TOPIC: (“oncolytic virus”) or TOPIC: (“oncolytic virotherapy”) or TOPIC: (“oncolytic viral therapy”) or TOPIC: (“anti cancer virus”) or TOPIC: (“anti tumor virus”). The language of publication was limited to English.

### 2.2 Data collection

The data of all publications that met the criteria was extracted on a single day, December 31, 2018. The data downloaded from the WoSCC were analyzed quantitatively and qualitatively by Microsoft Excel 2016 (Redmond, WA) and CiteSpace V (Philadelphia, PA). CiteSpace V software was used for raw data analysis and visualization, which was developed at Drexel University (Philadelphia, PA) for data analysis.^[10,11]^

### 2.3 Data analysis and interpretation

We built 4 analytic models to predict the time tendency of changes in publication trends, including linear model, exponential model, logarithmic model, and polynomial model. Considering a larger R^2^ represents a more optimal fitness. Therefore, we chose y = 0.0643x^2^ − 247.95x + 238804 as a more reasonable model to predict the time tendency of changes in publication trends as a growth model, in which “x” is “publication year: 2000 to 2018” and y is “cumulative papers by year” (Fig. S1).

In addition to the number of publications over time, the main data retrieved and analyzed for trends were related to journal name and impact factor (IF), journal subject area, country or region, institution, author, funding resources, and keywords or reference analysis by topic. All analysis of publications was based on CiteSpace V. The software selected the corresponding publication of each time to create a type of node and the visualization was presented as Cluster-View-Static and Show-Merged-Network.

## 3 Results and discussion

### 3.1 Global trend of publications on OVs

**Figure 2:**
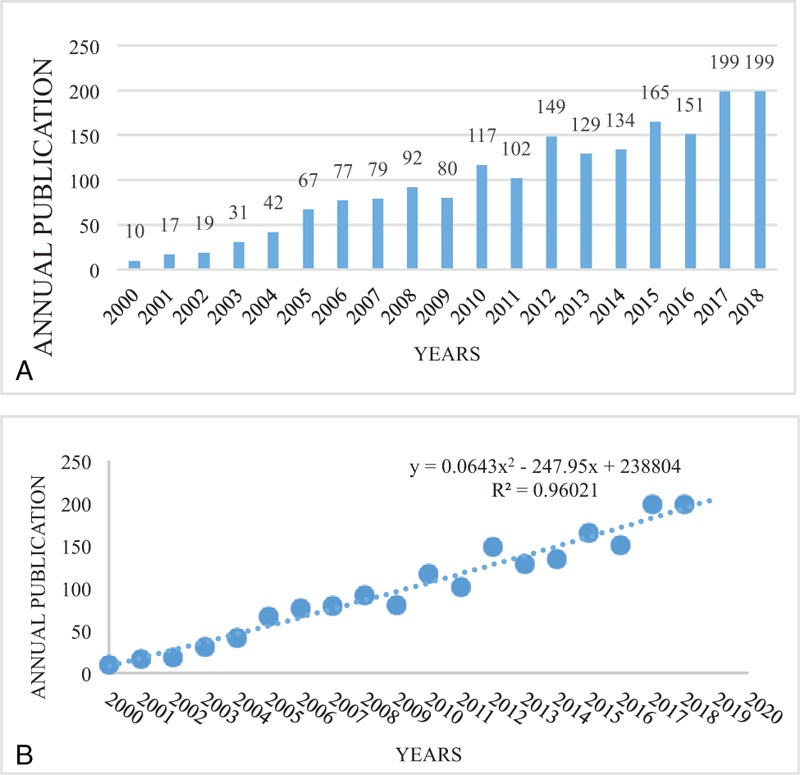
Distribution of (A) yearly publications on oncolytic virotherapy from 2000 to 2018, and (B) the model-fitting curve for the growth tendency of oncolytic virotherapy-related publications.

According to the literature inclusion procedure (Fig. 1), 1859 publications met the search criteria. The distribution of yearly publications and the growth trend of the model-fitting curve (R^2^ = 0.9602) are shown in Figure 2A and B. Indeed, the publishing trend showed a massive amount of change in the last decade, increasing from only 10 publications in 2000 to 199 publications in 2018. The continuing activity of this research reveals that OVs have gradually entered the horizon in the oncology field. Moreover, the growth trend model predicted that 303 papers on OVs will be published in 2019, exhibiting the growing passion of researchers in this field.

Thus, our analysis clearly demonstrates the rapid expansion of global OV research in the last decade in a quantitative manner. One of the key reasons for this trend might be related to the increasing prevalence of cancer associated with the rising proportion of the geriatric population and changing lifestyles in developing economies.^[12]^ The rapid rise in the number of cancer cases worldwide has further motivated research to meet the clinical demand for improved diagnostic and prevention methods of cancer. This in turn stimulates the research and development of alternate therapies by academic institutes as well as pharmaceutical companies for establishing novel cancer treatment strategies with high potency and low toxicity compared to conventional modalities.

### 3.2 Contributions of countries/regions to OV research

**Figure 3:**
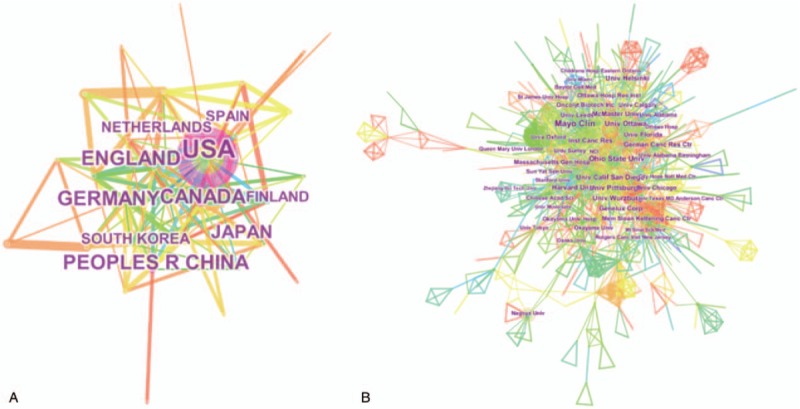
(A) Countries/territories and (B) institutions focusing on oncolytic virotherapy research.

As shown in the Table 1, the United States clearly dominates the number of publications in this field (981, 52.770%), followed by Canada (244, 13.125%) and China (205, 11.027%). A network map was created using CiteSpace to visualize the geographical distribution of countries or territories contributing to the field of OVs, which is presented in Figure 3A, matching the general ranking trends in Table 1. Although China ranked third in number of publications, the overall performance in terms of total citations and the H-index was inferior to that of the UK and Germany.

**Table 1:**
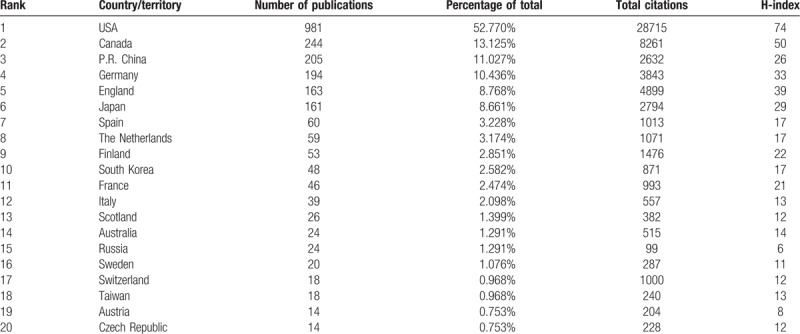
Distribution of publications in the top 20 countries/regions.

Thus, more than half of the top 20 institutes contributing to this field are located in the USA, suggesting that American scientists have taken the leading position in this field. Indeed, this has been accompanied by huge benefits to the population. In particular, the most successful oncolytic virotherapy agent T-VEC has revolutionized the field of cancer treatment and resulted in a significant improvement in durable responses for patients with melanoma.

### 3.3 Contributions of journals to OV research

**Figure 4:**
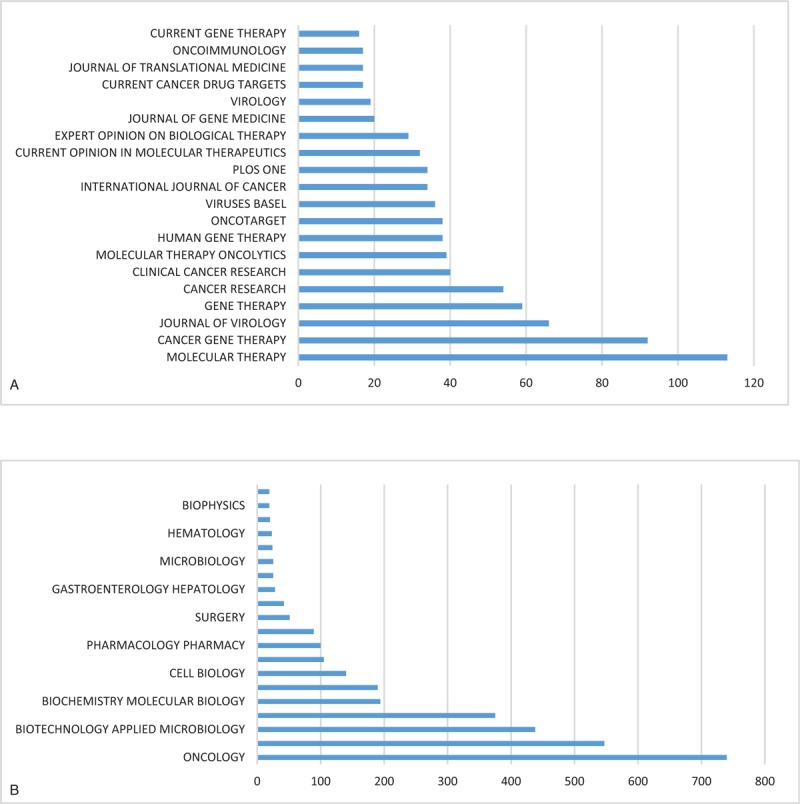
Distribution of the (A) top 20 journals and (B) research areas publishing oncolytic virotherapy research.

As shown in Figure 4A, the top 15 academic journals account for over one-third of the total publications on OVs research (724, 38.95%). *Molecular Therapy*, whose IF in 2017 was 7.008, ranked first in publications in this field (113, 6.079%), followed by *Cancer Gene Therapy* (92, 4.949%). Figure 4B showed the top 20 research hotspots that journals focused on. It revealed that oncology ranked first in the research hotspot of OVs research, with 740 publications. Other hot research hotspots included research experimental medicine, biotechnology applied microbiology and genetics heredity.

**Figure 5:**
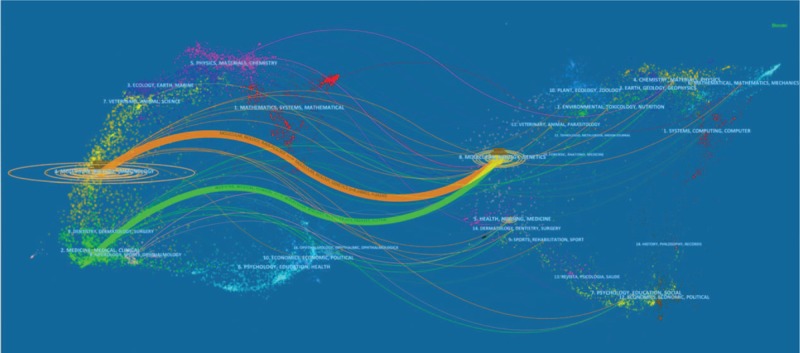
Dual-map overlay of journals publishing work related to oncolytic viruses.

Figure 5 shows a dual-map overlay of the number of papers with respect to the type or focus of the journal. Overall, journals with a focus in the fields of medicine, clinical, molecular biology, and immunology published the most papers on OVs, whereas the most highly cited papers were published in nursing, molecular biology, genetics, health, and medicine journals. Regarding the top 15 journals, *Clinical Cancer Research* had the highest IF in 2017 (10.199), and three others had an IF between 7 and 10, including *Molecular Therapy* (7.008), *Cancer Research* (9.130), and *International Journal of Cancer* (7.360). Overall, *Molecular Therapy* was the journal that contributed the most articles on OVs in the last decade, and given its reliable content, we can expect future breakthroughs in this field to be published there.

### 3.4 Distribution of institutions with research groups focusing on OVs

As shown in Figure 3B, over 1300 institutions have groups engaged in OVs research, and cooperation among mainstream institutions is frequent. The top 20 institutes contributing to OVs research are listed in Table 2. The Mayo Clinic accounted for 7.154% of total publications in this period, followed by University of Ottawa (5.325%) and University of California System (4.895%). In line with the regional analysis, institutes in the USA accounted for over half of the top 20 institutions (13/20), and the others are scattered across Canada, the UK, Germany, and Finland. Thus, although there is no doubt that North America currently has the most powerful impact on the field with respect to both productivity and contribution, European institutions also play an outstanding role.

**Table 2:**
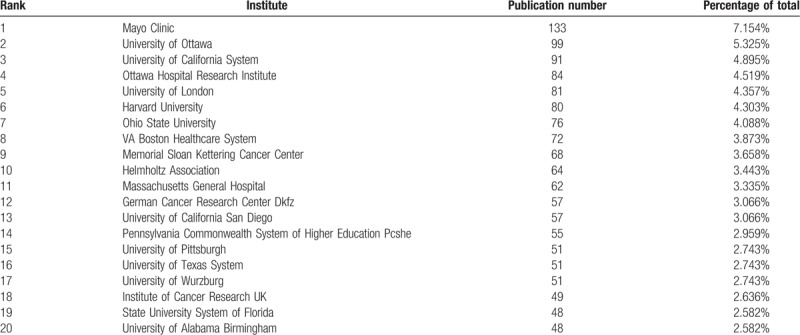
Top 20 institutions contributing to publications on oncolytic virotherapy research.

### 3.5 Contributions of authors to OV research

**Figure 6:**
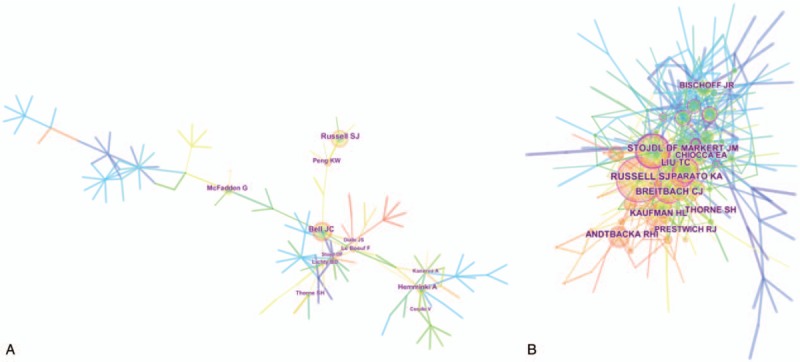
Authors working in the field of oncolytic virus (OV) research. (A) Active authors focused on OVs research. (B) Co-cited authors focused on OVs research.

Table 3 lists the top 10 authors that are involved in the field of OV research, who have collectively published a total of 417 papers, accounting for 22.4% of all published papers in the field. The co-authors map (Fig. 6A), generated by CiteSpace, displays the degree of cooperation between authors. JC Bell (65 publications) was identified as the most active author in the field, followed by SJ Russel (54 publications) and AA Szalay (45 publications). Author citations are also an important method to estimate the scientific relevance of publications. As shown in Figure 6B, the top 3 co-cited authors were SJ Russel, CJ Breitbach, and DF Stojdl.

**Table 3:**
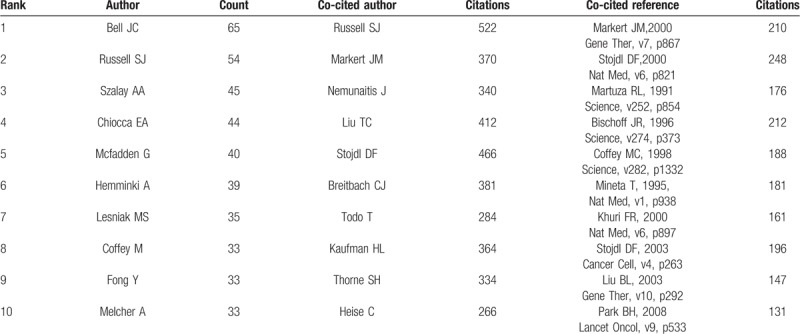
Top 10 authors, co-cited authors, and co-cited references on oncolytic virotherapy research.

Bell et al^[13,14]^ first tried to use vesicular stomatitis virus as a potent systemic anti-cancer agent, and then focused on engineering an OV to promote the virus's effect.^[15,16]^ Szalay et al^[17–19]^ have emphasized the use of recombinant viruses as cancer vaccinia. Thus, these scientists are currently the leaders in OV research, and their pioneering studies may still be instructive for future experimental designs.

### 3.6 Reference analysis on OVs

**Figure 7:**
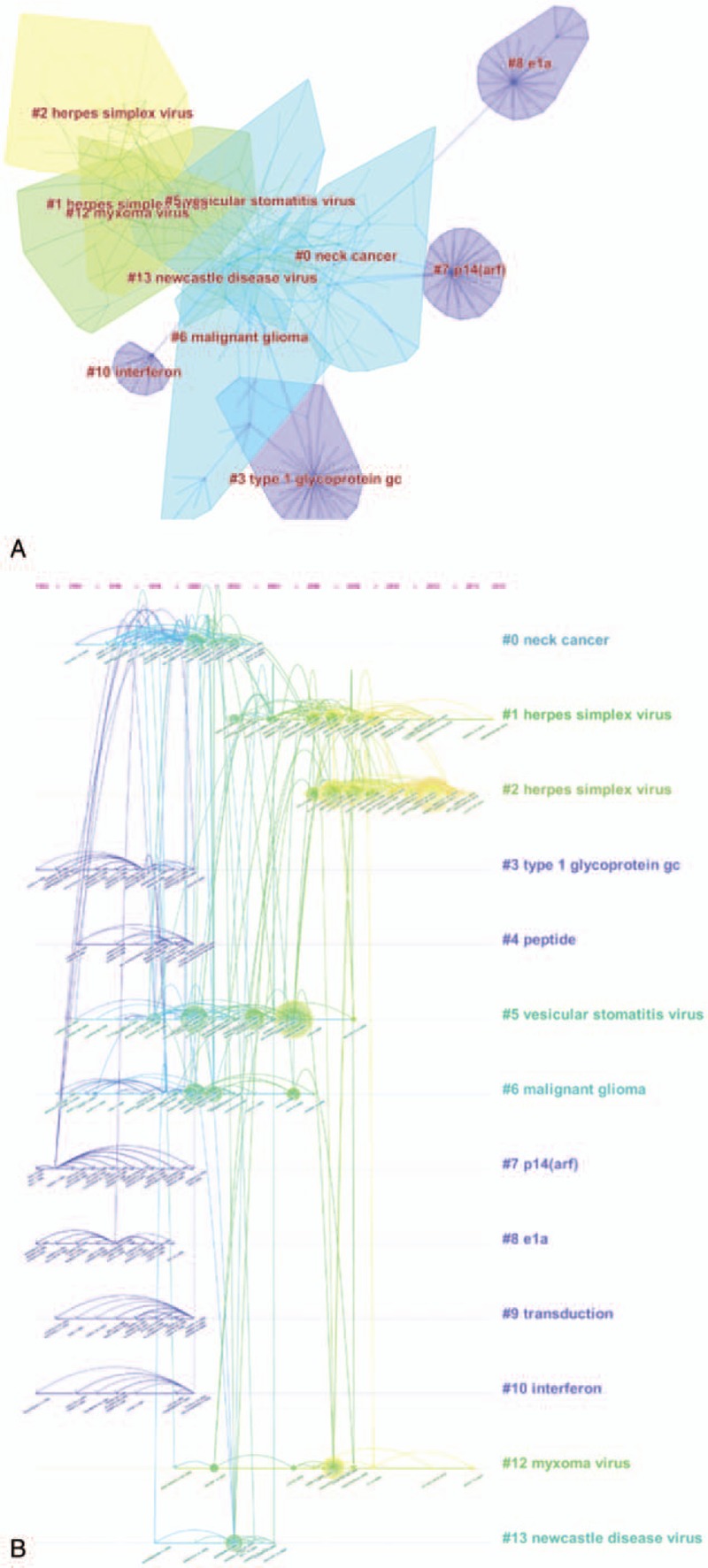
Reference analysis. (A) Co-citation map of references from publications on oncolytic virus (OV) research. (B) Co-citation map (timeline view) of references from publications on OV research.

One of the core indexes of bibliometrics is reference analysis. A cited reference co-citation map was generated by CiteSpace V. As shown in Figure 7A, 13 clusters were generated in terms of topics in the field, and each cluster highlighted the high citation, research field and core literature group in a period, showing a distinct specialty or a thematic concentration. Among the 13 clusters, the largest cluster (#0) was related to neck cancer, followed by clusters #1 and #2 (herpes simplex virus) and cluster #3 (type 1 glycoprotein). The cluster of herpes simplex virus, which was concentrated between 2004 and 2014, occupied 2 spots of the top 4 largest clusters, showing that the virus was of great importance in the field. A timeline view map of all clusters was shown in Figure 7B, revealing the progress of the field and the development tendencies. This map also indicated that most of the clusters were concentrated between 2000 and 2012. Top 10 most co-cited references were showed in Table 3. Those publications laid the foundation of the filed, among which the article STOJDL DF published in Nature Medicine had the highest value of citations (248 citations).

### 3.7 Analysis of keywords related to OV research

**Figure 8:**
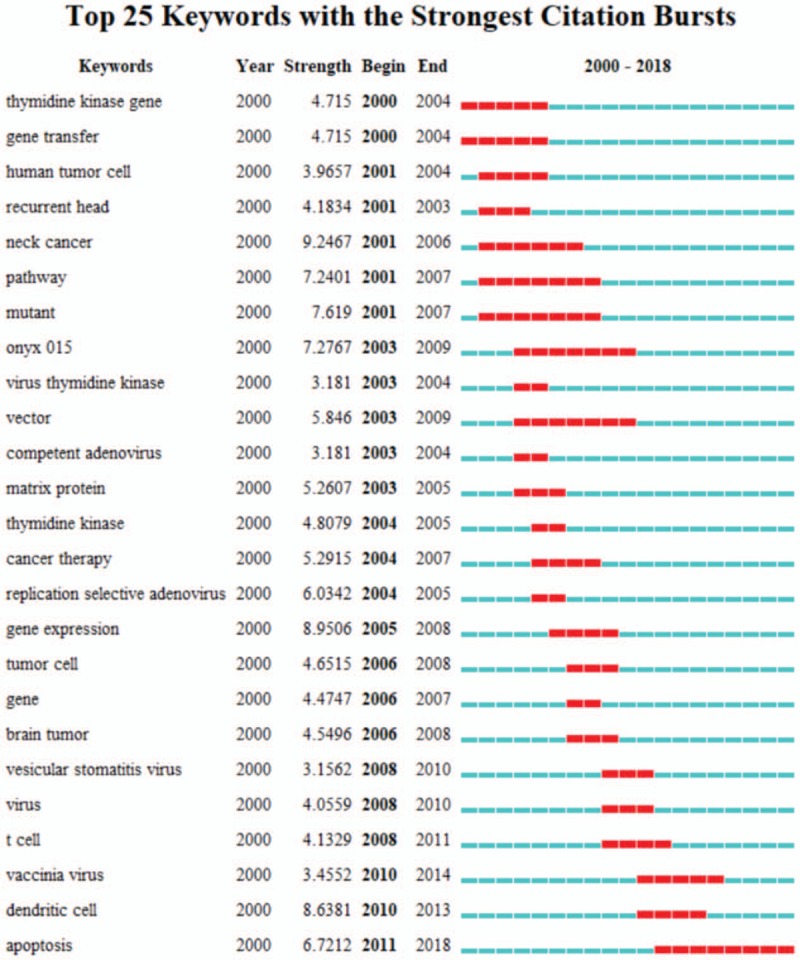
Top 25 keywords related to oncolytic virus research. The green line indicates time intervals, and the red line shows periods of burst.

Keywords can also serve as an important index to reflect research hotspots at a certain time, and burst words identified among all keywords in relevant publications can help to predict new frontier topics. Thus, through analyzing the most common keywords of articles, we can start to understand the development of a research topic in a more comprehensive manner.^[11]^ As shown in Figure 8, the keyword with the strongest citation strength was “neck cancer” (9.2467), and this trend lasted for five consecutive years (2001–2006). In 2001, Nemunaitis et al^[20]^ reported a phase II trial of the intratumoral administration of ONYX-015, a replication-selective adenovirus, in patients with refractory head and neck cancer, which suggested evidence for modest antitumoral activity. In 2006, China approved the world's first OV therapy for head and neck cancer.^[21]^ The other 2 strongly cited keywords were “gene expression” (8.9506, 2005–2008) and “dendritic cell” (8.6381, 2010–2013). Moreover, the results in Figure 8 indicate that an ongoing top hotspot in this field is apoptosis (6.7212, 2011–2018). This trend is related to a paper published by Mansour et al^[22]^ in 2011 demonstrating that the oncolytic selectivity of Newcastle disease virus for tumor cells was dependent on the extent of tumor cell resistance to apoptosis.

**Figure 9:**
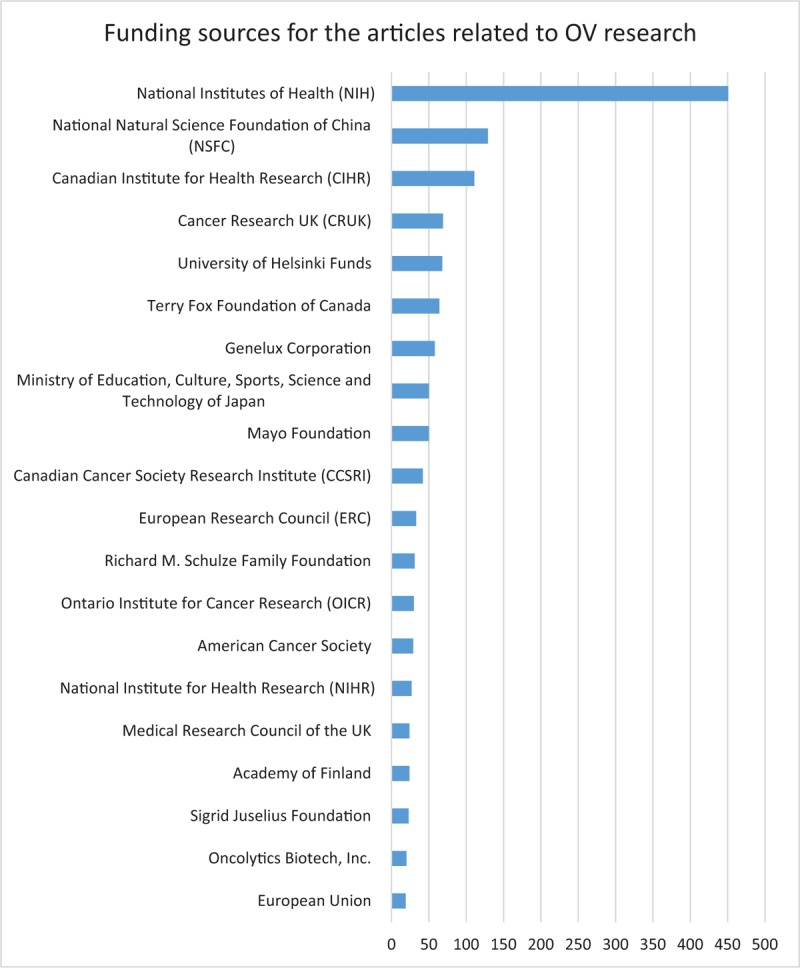
Top 20 funding sources for the articles related to OV research.

These shifts in trends in topics and keywords demonstrate that the hotspots of research have changed over the past decade, from focus on neck cancer and gene expression to more recent exploration of the study of T cells, dendritic cells, and apoptosis (Fig. 9). This shift further highlights the recognition of the power of OVs along with parallel research progress on tumor immune responses. On the one hand, localized OV treatment can kill cancer cells to cause the release of tumor associated antigens (TAAs), which prime tumor-specific T cell responses in the draining lymph nodes. For “cold tumors”, the new antigen produced by the virus after lysing tumor cells can significantly increase the immunogenicity of the tumor. This is an important process to break this immunosuppressive environment, which is highly inhibited, and to induce cytotoxic T cells to produce local or systemic anti-tumor immune responses.^[23]^ On the other hand, OVs can also increase the T cell infiltration of cancer in multiple ways. Related studies have found that oncolytic viruses have strong neutrophil recruitment ability, and the recruited neutrophils can produce a series of inflammatory mediators and a large number of proteases with extracellular matrix-degrading ability, thereby promoting T cells infiltration.^[24]^ Therefore, a multi-drug combination strategy based on OVs to break the immunosuppressive environment and activate the patients’ own immune systems shows good prospects.

### 3.8 Funding sources for the articles related to OV research

The top 20 funding sources for the articles related to OV research were depicted in Figure 9. Considering the integrity of the data, we only retrieved and analyzed the articles published from 2008 to 2018. Almost one third of the articles (451, 29.794%) were funded by the National Institutes of Health (NIH). Other funding sources within the US, such as Genelux Corporation (58, 3.825%), Mayo Foundation (50, 3.298%), Richard M. Schulze Family Foundation (31, 2.044%), American Cancer Society, and National Institute for Health Research (NIHR) (27, 1.781%) were also the leading funders in the field. All funds provided by these organizations fueled the research of OV and this may explain why the US was the leading country in the field. National Natural Science Foundation of China (NSFC) (129, 8.509%) and Canadian Institute for Health Research (CIHR) (111, 7.322%) were also the major founders. Canada was obviously more enthusiastic about OV research. Besides CIHR, there were also a number of other foundations supporting this field, such as Terry Fox Foundation of Canada (64, 4.221%), Canadian Cancer Society Research Institute (CCSRI) (42, 2.770%) and Ontario Institute for Cancer Research (OICR) (30, 1.978%). Canadian scientist JC Bell (65 publications) was identified as the most active author in the field. European foundations such as Cancer Research UK (CRUK) (69, 4.551%), University of Helsinki Funds (68, 4.485%), European Research Council (ERC) (33, 2.176%), Medical Research Council of the UK (24, 1.583%), and Academy of Finland (24, 1.583%) contributed a lot to the field in terms of the number of the articles.

## 4 Conclusion

With the increasing number of elderly people worldwide accompanied by changing lifestyles, the global incidence of cancer is on the rise. Research on OVs has grown rapidly over the past decade, and OVs are now recognized as an important “weapon” for oncologists to control and destroy cancer.

Although the data analysis in this study is based on a relatively objective and comprehensive strategy, limitations are inevitable. Importantly, we only included English languages papers, which could have eliminated some key research published on OVs. However, Web of Science represents the largest bibliometric database currently available. In addition, papers published in 2019 were not analyzed. In future work, we will consider incorporating studies published in other languages along with updated research.

Nevertheless, considering all of the data obtained, we can conclude that more and more researchers and clinicians have started to pay attention to the potential of OVs in recent years, especially with respect to their effects on T cells, dendritic cells, and apoptosis. Thus, the high potency and low toxicity of OVs compared to other conventional remedies may offer a new and promising direction to achieve a cure for cancer. By comprehensively and quantitatively summarizing the trends in this field, we expect this work to help further advance the OV research agenda, and serve as a guide for focusing on the key trends.

## Author contributions

**Conceptualization:** Guowei Tan, chenghao huang.

**Data curation:** Yidi Zou.

**Formal analysis:** Yidi Zou, Jun Zhang, Ningshao Xia.

**Funding acquisition:** chenghao huang.

**Investigation:** Yong Luo.

**Methodology:** Yidi Zou, Yong Luo.

**Project administration:** chenghao huang.

**Software:** Yidi Zou.

**Visualization:** Yidi Zou.

**Writing – original draft:** Guowei Tan, chenghao huang.

**Writing – review & editing:** Jun Zhang, Ningshao Xia, chenghao huang.

## Supplementary Material

Supplemental Digital Content
