# Supplementary material for: Bibliometric analysis of oncolytic virus research, 2000 to 2018
Source: Medicine (Baltimore). 2019 Aug 30;98(35):e16817. doi: 10.1097/MD.0000000000016817 (PMC6736135; doi:10.1097/MD.0000000000016817)

Figure S1

Fig. S1 The model-fitting curves for the growth tendency of oncolytic virotherapy-related publications by using different analytic models. (A) Linear model. (B) Exponential model. (C) Logarithmic model. (C) Polynomial model.

A


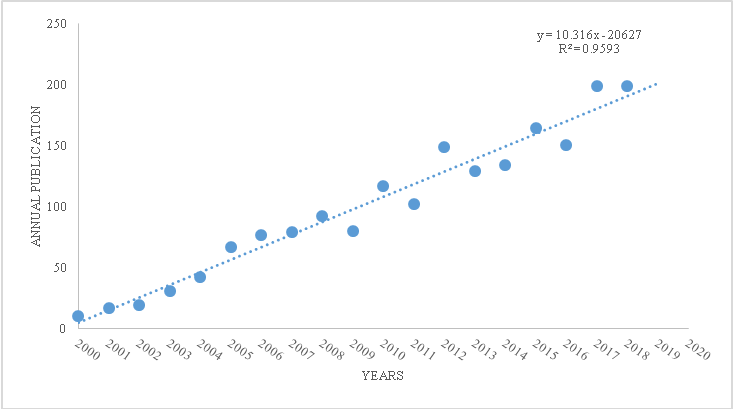


B


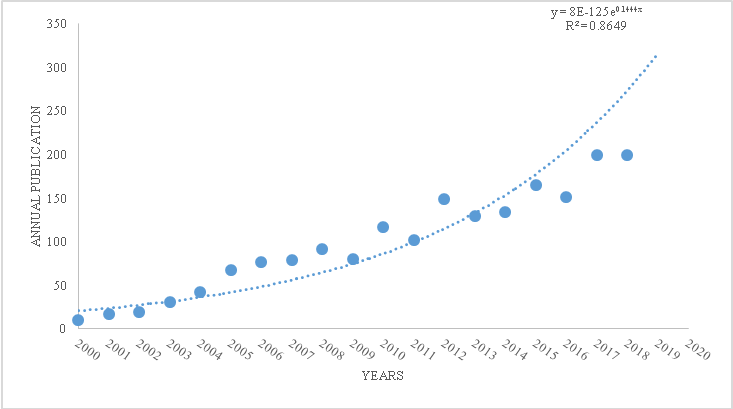


C


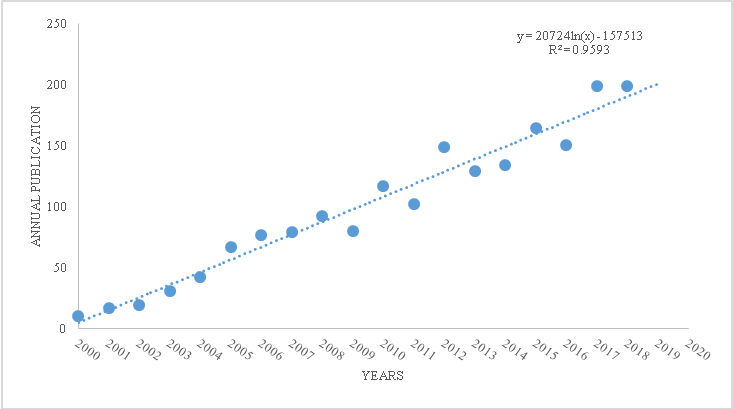


D


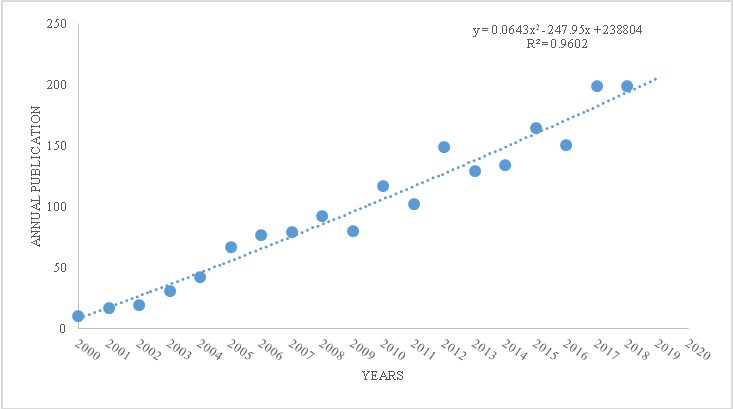

Supplement: Supplemental Digital Content [file medi-98-e16817-s001.docx]
